# Comparison of transdermal and oral anti-inflammatory drugs on immediate post-operative pain control in total knee arthroplasty: a randomized controlled trial

**DOI:** 10.1186/s42836-026-00395-6

**Published:** 2026-06-08

**Authors:** Nonn Jaruthien, Rachata Piriyamanun, Srihatach Ngarmukos, Aree Tanavalee, Virinaree Kampitak, Chotetawan Tanavalee, Chavarin Amarase

**Affiliations:** 1 Biologics for Knee Osteoarthritis Research Unit, Faculty of Medicine, Chulalongkorn University, Bangkok, 10330 Thailand; 2 Department of Orthopaedics, Faculty of Medicine and King Chulalongkorn Memorial Hospital, Chulalongkorn University, Bangkok, 10330 Thailand; 3 Department of Anaesthesiology, Faculty of Medicine and King Chulalongkorn Memorial Hospital, Chulalongkorn University, Bangkok, 10330 Thailand

**Keywords:** Nonsteroidal anti-inflammatory drugs, NSAIDs, Post-operative pain, Total knee arthroplasty, TKA, Transdermal, Esflurbiprofen, Celecoxib

## Abstract

**Background:**

Oral nonsteroidal anti-inflammatory drugs (NSAIDs) are commonly used as multimodal pain-control medications in total knee arthroplasty (TKA), while topical NSAIDs are commonly used for chronic pain conditions. There is limited evidence on whether topical NSAIDs could provide effective pain control after surgery. We compared the efficacy of topical and oral NSAIDs for immediate post-operative pain following TKA.

**Methods:**

This prospective, randomized, non-inferiority trial included 100 patients undergoing primary TKA. Following uniform anesthesia and multimodal pain management, from post-operative day 1 (POD 1) onward, patients were randomly assigned to receive either a transdermal Esflurbiprofen patch (Group A) or oral Celecoxib (Group B) once daily for 14 days. Pain at rest and on motion, functional outcomes, and adverse events were serially evaluated from POD 0 through 6 weeks post-operatively.

**Results:**

There were 50 patients in both Group A and B, with a mean age of 72 years and a female predominance (over 70%). There were no differences in demographic data. Both groups had significantly lower visual analog scale (VAS) scores at rest and on motion at POD 0, POD 1, POD 2, POD 3, POD 5, POD 14, and the 6th week, with no differences between groups. The timed up-and-go test (TUGT), 5-time sit-to-stand test (5TSST), and 3-min walk test (3MWT) were similarly significantly worse in both groups at POD 14 and improved in the 6th week. The Western Ontario and McMaster Universities Osteoarthritis Index (WOMAC) was similarly improved at POD 3, POD 14, and in the 6th week, without differences. Both groups had no serious adverse events and no changes in creatinine clearance at any follow-up point.

**Conclusion:**

A multimodal pain control regimen in TKA using a transdermal Esflurbiprofen patch provided immediate post-operative pain control that was non-inferior to oral Celecoxib, with comparable VAS, functional outcomes, and safety profiles.

## Introduction

Total knee arthroplasty (TKA) is an optimal treatment for advanced knee osteoarthritis (KOA), providing substantial pain relief, cost-effectiveness, and significant improvements in quality of life [1]. Regarding multimodal pain control in TKA, it combines different analgesic modalities and multiple nerve blocks with different mechanisms of action, achieving superior pain control, minimal opioid consumption, and reduced treatment-related adverse effects, thereby facilitating functional recovery [2, 3]. Nonsteroidal anti-inflammatory drugs (NSAIDs), including cyclo-oxygenase-2 (COX-2) inhibitors, play an important role in multimodal pain management during the peri-operative period and for several weeks after discharge. Besides enhancing the effects of other painkillers to reduce pain at rest, the anti-inflammatory effects of NSAIDs help reduce pain during movement and facilitate early rehabilitation when administered post-operatively [4].

Although the current peri-operative pain control of TKA during patient admission was effective, the acute post-operative pain after hospital discharge remains an unresolved issue [5]. Insufficient pain control after discharge is associated with multiple adverse outcomes during follow-up (FU), including delayed progress of mobilization and knee range of motion, reduced satisfactory level after surgery, increased risk of venous thromboembolism, and a risk for persistent long-term post-operative pain [6–9]. Therefore, oral NSAIDs are one of the common medications prescribed to TKA patients at discharge, of which oral COX-2 inhibitors are the preferred choice. However, patients are instructed not to use long-term NSAIDs to avoid adverse effects, such as myocardial ischemia, renal dysfunction, and gastrointestinal ulceration [10–12].

Besides the oral form, other forms of NSAIDs are available in the market, which are claimed to reduce their adverse events. Recently, transdermal NSAIDs have emerged as an alternative analgesic option for the treatment of KOA, offering localized anti-inflammatory and analgesic effects with minimal systemic absorption. This route of administration may reduce the risk of systemic adverse effects associated with oral NSAIDs while providing effective pain control [13–15].

Although several studies have proven the efficacy of transdermal NSAIDs in KOA [16–18], the established role for their efficacy in post-operative pain control and safety after TKA is limited. Therefore, the present study aimed to compare the efficacy of a transdermal NSAID (Esflurbiprofen transdermal patch) with an oral COX-2 inhibitor (Celecoxib) in managing perioperative and post-discharge pain after TKA.

## Materials and methods

### Study design

This study was conducted as a single-center, prospective, randomized controlled trial (RCT). The trial was approved by the Chulalongkorn University Institutional Review Board (IRB) (COA No. 1363/2024 and IRB No. 1363/2024) and was registered at thaiclinicaltrials.org (TCTR20250625003). The study was conducted from November 2024 to October 2025. All methods and experimental protocols were carried out in accordance with relevant guidelines and regulations. Informed consent was obtained from all patients who participated in the study.

### Patient selection

Inclusion criteria were age ≥ 50 years and a diagnosis of KOA requiring primary unilateral TKA. Exclusion criteria included prior surgery on the same knee, history of lower extremity surgery within the previous 6 months, radiographic atherosclerosis of the femoral artery, use of NSAIDs within 2 weeks prior to surgery, known hypersensitivity to study medications, severe gastrointestinal disease, severe medical comorbidities limiting the ability to perform moderate-intensity aerobic exercise, major neurological conditions, and inability to provide informed consent or reliable medical history.

### Randomization and intervention

**Fig. 1:**
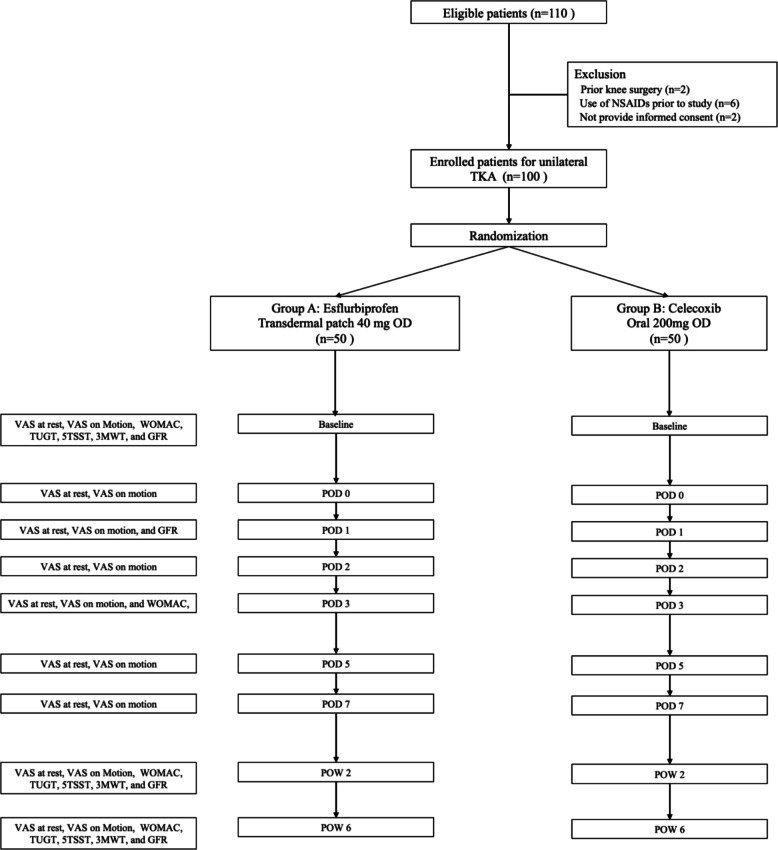
CONSORT flow diagram of the study. VAS: visual analog scale; WOMAC: Western Ontario and McMaster Universities Osteoarthritis Index; POD: postoperative day; POW: postoperative week; GFR: glomerular filtration rate; TUGT: time-up-and-go test; 5TSST: five-time sit-to-stand test; 3MWT: three-minute walk test

After pre-operative assessment, 110 eligible participants undergoing primary unilateral TKA were enrolled in the study. Ten patients were excluded due to prior surgeries, use of NSAIDs within 2 weeks prior to surgery, and failure to provide informed consent. The remaining 100 patients were randomly assigned to receive either a transdermal 40 mg Esflurbiprofen patch (Locoa, Taisho Pharmaceutical, Tokyo, Japan) or an oral 200 mg Celecoxib (Celebrex, Viatris, Canonsburg, PA, USA) as part of post-operative multimodal pain management after TKA. The randomization was performed using stratified block randomization with a 1:1 allocation ratio. A computer-generated randomization sequence was used, and allocation concealment was ensured by sealed envelopes prepared by an independent research assistant, which were opened after surgery. The Consolidated Standards of Reporting Trials (CONSORT) study flow diagram is shown in Fig. 1. The baseline demographic and clinical characteristics of the participants are summarized in Table 1.

**Table 1:** Demographic data of the studied group

Demographic data	Group A	Group B	*p*-value
	** Esflubiprofen**	**Oral celecoxib**	
	**(*N* = 50)**	**(*N* = 50)**	
Sex-Female (N, %)	43 (86.0%)	37 (74.0%)	0.21
Age: year (mean, SD)	71.9 (7.0)	72.3 (7.1)	0.78
Weight: Kg (mean, SD)	69.3 (13.6)	65.3 (11.3)	0.11
Height: cm (mean, SD)	154.7 (6.4)	156.6 (8.6)	0.22
BMI (mean, SD)	28.6 (5.4)	26.7 (4.8)	0.067
ASA class 2 (N, %)	28 (56.0%)	34 (68.0%)	0.3
ASA class 3 (N, %)	22 (44.0%)	16 (32.0%)	
Legion total knee system (N, %)	32 (64%)	33 (66%)	0.95
Attune total knee system (N, %)	28 (36%)	27 (34%)	
Tourniquet time: min (mean, SD)	85 (15)	86 (14)	0.88

### Anesthesia, surgery, and post-operative pain management

All procedures were performed under general anesthesia by 2 senior surgeons (AT and SN) using a mini-midvastus approach with the concept of restoring neutral mechanical alignment. Using manual instruments for all bone cuts, a single surgical technique was applied, including posterior referencing systems for femoral sizing, fixed 3-degree femoral external rotation, the medial one-third of the tibial tubercle as the reference for tibial component rotation, and onset resurfacing of the patella with a tourniquet pressure of 320 mmHg inflated before skin incision and deflated after wound closure. Two posterior stabilized total knee systems (LEGION, Smith & Nephew, Memphis, TN, USA, and ATTUNE, DePuy Synthes, New Brunswick, NJ, USA) were used with cemented fixation (PALACOS, Heraeus, Wehrheim, Germany) in all knees. One gram of tranexamic acid was injected intra-articularly after wound closure, and no vacuum drain was inserted.

Post-operatively, all patients underwent continuous adductor canal block (cACB) in combination with local anesthetic infiltration into the interspace between the popliteal artery and posterior capsule of the knee (iPACK) as part of an opioid-sparing multimodal analgesic protocol [19, 20]. The adductor canal block was initiated with 15 mL of 0.25% levobupivacaine, followed by continuous infusion of 0.15% levobupivacaine at a rate of 5 mL/h via a disposable infusion pump for 48 h [21]. A uniform multimodal analgesic regimen was administered to all patients, consisting of oral paracetamol (500 mg every 6 h) and pregabalin (75 mg once daily at bedtime), except for randomization to receive one of the two NSAIDs. Breakthrough pain was managed with intravenous morphine (3 mg every 3 h as needed) for patients reporting a visual analog scale (VAS; 0–10) score of 5 or higher during the first 48 h post-operatively.

Post-operatively, patients received either a transdermal Esflurbiprofen patch or oral Celecoxib once daily for 14 days, starting on post-operative day 1 (POD 1). In the Esflurbiprofen group (Group A), the patch was applied to the anteromedial knee of the operated limb after the patient had breakfast. The patch application area was changed if any local skin irritation occurred. Any local or systemic reactions led to the patient’s concern, the Esflurbiprofen patch was discontinued, and the patient was withdrawn from the study. In the Celecoxib group (Group B), patients received 200 mg of oral Celecoxib once daily for 14 days after breakfast. Similarly, any adverse events led the patient to discontinue Celecoxib, and the patient was withdrawn from the study.

### Outcome measures

All patients were evaluated for VAS at rest and during movement at baseline, post-operative day (POD) 0, POD 1, POD 2, POD 3, POD 5, POD 7, POD 14, and 6 weeks post-operatively. The primary outcomes were between-group differences in pain intensity, measured using the VAS at rest and during movement on POD 14. Secondary outcomes included between-group differences in VAS at rest and during movement at 6 weeks post-operatively. Knee function was assessed using performance-based measures (PBMs) at baseline, POD 14, and 6 weeks post-operatively, including the 5-time sit-to-stand test (5TSST), the timed up-and-go test (TUGT), and the 3-min walk test (3MWT). Similarly, patient-reported outcome measure (PROM), including the Western Ontario and McMaster Universities Osteoarthritis (WOMAC) index, was collected at baseline, POD 3, POD 14, and 6 weeks post-operatively. The glomerular filtration rate (GFR) of both groups was evaluated at baseline, POD 14, and 6 weeks post-operatively. The incidence of adverse drug events was recorded throughout the study.

### Sample size and statistical analysis

The sample size was calculated to test the non-inferiority of Esflurbiprofen compared with Celecoxib using VAS on POD 14, assuming equal variances between groups. According to the study by Gong et al., the Celecoxib group had a mean VAS of 16.4 (SD, 17.1), and a minimal clinically important difference (MCID) for the VAS was defined as 11.5 points on a 0–100-point scale and was used to determine the non-inferiority margin, which was set at 1.15 on a 0–10 scale [22]. A one-sided significance level (α) of 0.025 and a statistical power of 80% (β = 0.20) were applied. Sample size estimation was calculated using the two-sample independent means formula in Stata MP version 18.0 (StataCorp, College Station, TX, USA). A sample size of 44 participants per group was required. To account for an anticipated 10% dropout rate, the final sample size per group was increased to 49, for a total of 98 participants.

Baseline characteristics were presented as mean ± standard deviation (SD) for continuous variables and as frequencies and percentages for categorical variables. Outcomes were reported as mean ± SD with 95% confidence intervals (95% CI). The primary non-inferiority analysis compared mean VAS between both groups on POD 14 using an independent t-test, with non-inferiority concluded if the upper bound of the 95% CI for the between-group difference did not exceed the prespecified margin of 1.15 points. Secondary outcomes, including VAS, TUGT, 5TSST, 3MWT, and WOMAC index, were compared between groups at each time point using two-way repeated-measures analysis of variance (ANOVA). Between-group comparisons of post-operative glomerular filtration rate (GFR) at each time point were performed using ANOVA.

## Results

All 100 participants completed all tasks at all evaluation points, with no dropouts. There were no statistically significant differences between Group A and Group B in demographic data, including gender distribution, age, body weight, height, body mass index (BMI), American Society of Anesthesiologists (ASA) classification, or tourniquet time (Table 1). However, more women were dominant in Group A (86.0%) than in Group B (74.0%), but the difference was not statistically significant (*p* = 0.21).

**Fig. 2:**
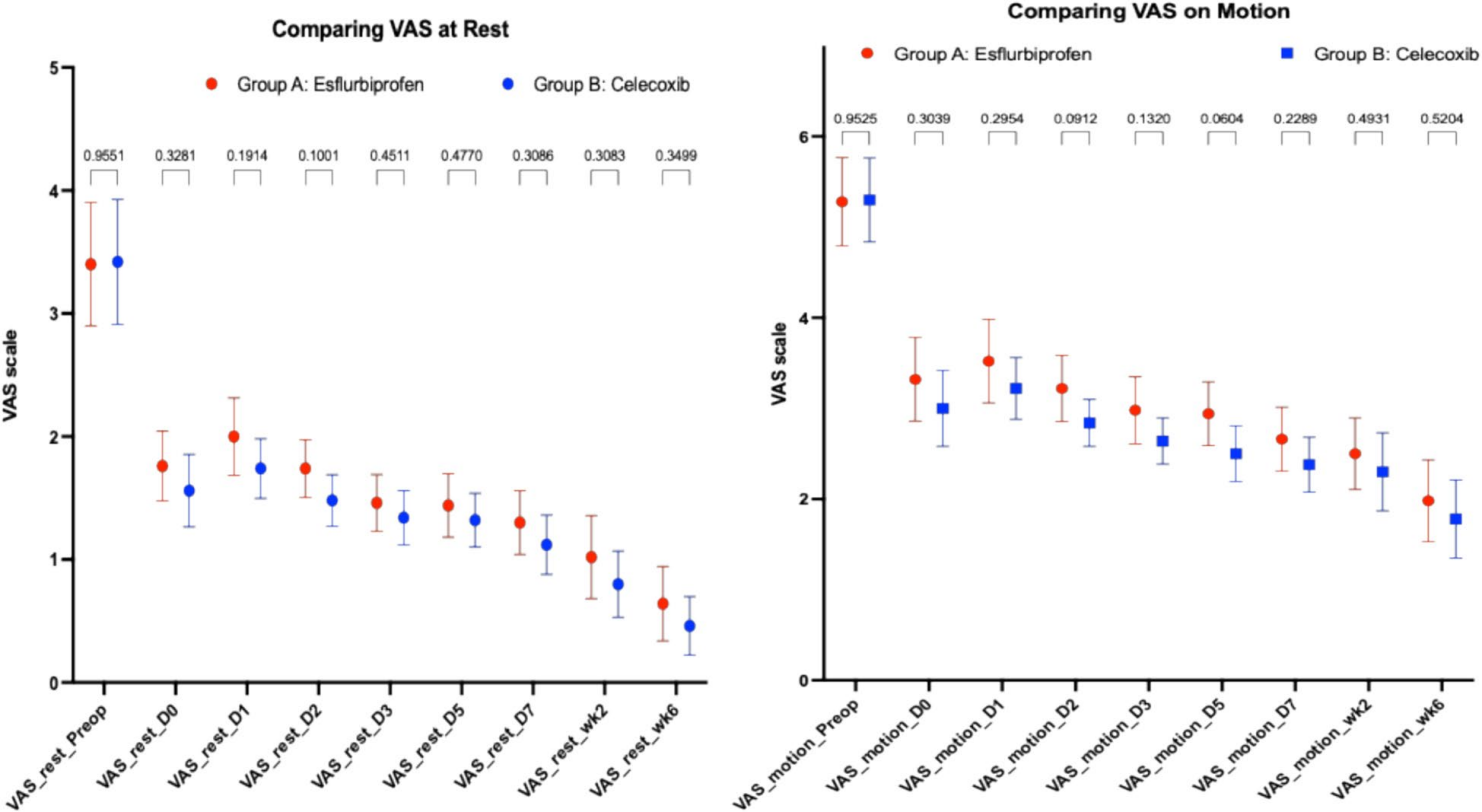
Comparing VAS at rest and on motion between Group A: Esflurbiprofen and Group B: Celecoxib. There were no differences in VAS improvement at rest or on motion at any evaluation point from baseline to 6 weeks postoperatively between the two groups

**Fig. 3:**
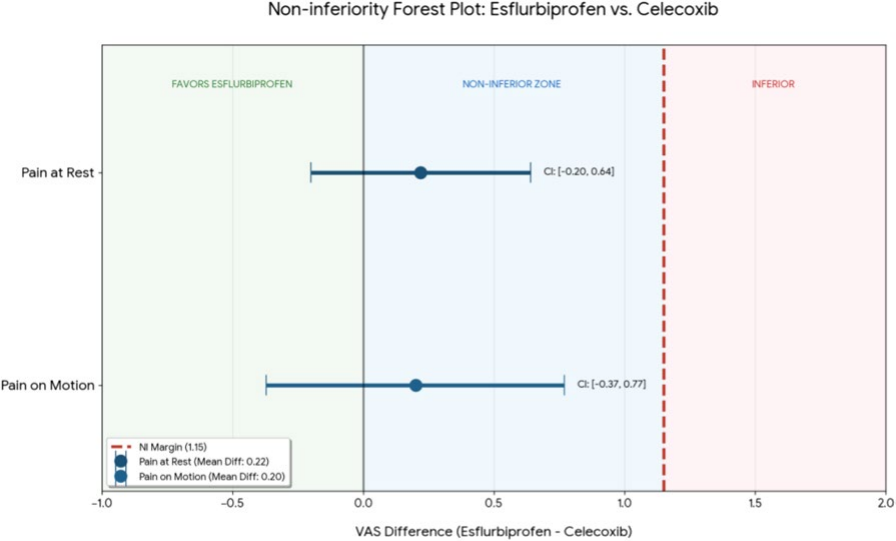
Graph with details in table demonstrating a non-inferiority forest plot in VAS difference levels at rest and on motion of Esflurbiprofen (Group A) and Celecoxib (Group B) on postoperative day 14, where the margin of VAS difference at rest and on motion was 1.15

In both groups, pain at rest and on motion decreased significantly throughout all FU points, as shown in Table 2. On POD 14, there were no statistically significant differences between Group A and Group B in VAS at rest (mean ± SD: 1.0 ± 1.2 vs 0.8 ± 0.9, *p* = 0.31) and on motion (mean ± SD: 2.5 ± 1.4 vs 2.3 ± 1.5, *p* = 0.49) as shown in Fig. 2. Although the mean VAS score in pain at rest and pain on motion of the Esflurbiprofen group were higher than those in the Celecoxib group, the between-group mean difference at postoperative day 14 was 0.22 (95% CI: − 0.20 to 0.64) for pain at rest and 0.20 (95% CI: − 0.37 to 0.77) for pain at motion. The upper bounds of both confidence intervals remained within the prespecified non-inferiority margin of 1.15 points, indicating that the efficacy of Esflurbiprofen for post-operative pain control following TKA was non-inferior to Celecoxib (Fig. 3). There were no patients in either group who needed morphine as pain relief.

**Table 2:** Changes in VAS at rest and on motion of both groups from baseline to the 6th week. Compared to baseline VAS, both groups had significantly improved VAS at rest and on motion from postoperative day 0 (POD 0) through POD 3, POD 5, the 2nd-week, and the 6th-week follow-ups

**Changes in Pain at Rest of Group A: Esflurbiprofen**									
**Parameter**	**VAS_r_Preop**	**VAS_r_D0**	**VAS_r_D1**	**VAS_r_D2**	**VAS_r_D3**	**VAS_r_D5**	**VAS_r_D7**	**VAS_r_wk2**	**VAS_r_wk6**
Minimum	1	0	0	0	0	0	0	**0**	0
Maximum	7	5	6	4	4	5	5	**5**	6
Range	6	5	6	4	4	5	5	**5**	6
Mean	3.4	1.8	2.0	1.7	1.5	1.4	1.3	**1.0**	0.6
Std. Deviation	1.8	1.0	1.1	0.8	0.8	0.9	0.9	**1.2**	1.1
Std. Error of Mean	0.2	0.1	0.2	0.1	0.1	0.1	0.1	**0.2**	0.2
Lower 95% CI	2.9	1.5	1.7	1.5	1.2	1.2	1.0	**0.7**	0.3
Upper 95% CI	3.9	2.0	2.3	2.0	1.7	1.7	1.6	**1.4**	0.9
*p*-value		< 0.0001	< 0.0001	< 0.0001	< 0.0001	< 0.0001	< 0.0001	** < 0.0001**	< 0.0001
**Changes in Pain at Rest of Group B: Celecoxib**									
**Parameter**	**VAS_r_Preop**	**VAS_r_D0**	**VAS_r_D1**	**VAS_r_D2**	**VAS_r_D3**	**VAS_r_D5**	**VAS_r_D7**	**VAS_r_wk2**	**VAS_r_wk6**
Minimum	0	0	0	0	0	0	0	**0**	0
Maximum	7	4	4	3	4	4	5	**4**	4
Range	7	4	4	3	4	4	5	**4**	4
Mean	3.4	1.6	1.7	1.5	1.3	1.3	1.1	**0.8**	0.5
Std. Deviation	1.8	1.0	0.9	0.7	0.8	0.8	0.8	**0.9**	0.8
Std. Error of Mean	0.3	0.1	0.1	0.1	0.1	0.1	0.1	**0.1**	0.1
Lower 95% CI	2.9	1.3	1.5	1.3	1.1	1.1	0.9	**0.5**	0.2
Upper 95% CI	3.9	1.9	2.0	1.7	1.6	1.5	1.4	**1.1**	0.7
*p*-value		< 0.0001	< 0.0001	< 0.0001	< 0.0001	< 0.0001	< 0.0001	** < 0.0001**	< 0.0001
**Changes in Pain on Motion of Group A: Esflurbiprofen**									
**Parameter**	**VAS_m_Preop**	**VAS_m_D0**	**VAS_m_D1**	**VAS_m_D2**	**VAS_m_D3**	**VAS_m_D5**	**VAS_m_D7**	**VAS_m_wk2**	**VAS_m_wk6**
Minimum	3	0	0	0	0	0	0	**0**	0
Maximum	9	7	9	7	6	5	5	**6**	6
Range	6	7	9	7	6	5	5	**6**	6
Mean	5.3	3.3	3.5	3.2	3.0	2.9	2.7	**2.5**	2.0
Std. Deviation	1.7	1.6	1.6	1.3	1.3	1.2	1.2	**1.4**	1.6
Std. Error of Mean	0.2	0.2	0.2	0.2	0.2	0.2	0.2	**0.2**	0.2
Lower 95% CI	4.8	2.9	3.1	2.9	2.6	2.6	2.3	**2.1**	1.5
Upper 95% CI	5.8	3.8	4.0	3.6	3.4	3.3	3.0	**2.9**	2.4
*p*-value		< 0.0001	< 0.0001	< 0.0001	< 0.0001	< 0.0001	< 0.0001	** < 0.0001**	< 0.0001
**Changes in Pain on Motion of Group B: Celecoxib**									
**Parameter**	**VAS_m_Preop**	**VAS_m_D0**	**VAS_m_D1**	**VAS_m_D2**	**VAS_m_D3**	**VAS_m_D5**	**VAS_m_D7**	**VAS_m_wk2**	**VAS_m_wk6**
Minimum	2	0	1	1	1	0	0	**0**	0
Maximum	9	5	5	4	4	4	5	**7**	6
Range	7	5	4	3	3	4	5	**7**	6
Mean	5.3	3.0	3.2	2.8	2.6	2.5	2.4	**2.3**	1.8
Std. Deviation	1.6	1.5	1.2	0.9	0.9	1.1	1.1	**1.5**	1.5
Std. Error of Mean	0.2	0.2	0.2	0.1	0.1	0.2	0.2	**0.2**	0.2
Lower 95% CI	4.8	2.6	2.9	2.6	2.4	2.2	2.1	**1.9**	1.4
Upper 95% CI	5.8	3.4	3.6	3.1	2.9	2.8	2.7	**2.7**	2.2
*p*-value		< 0.0001	< 0.0001	< 0.0001	< 0.0001	< 0.0001	< 0.0001	** < 0.0001**	< 0.0001

**Fig. 4:**
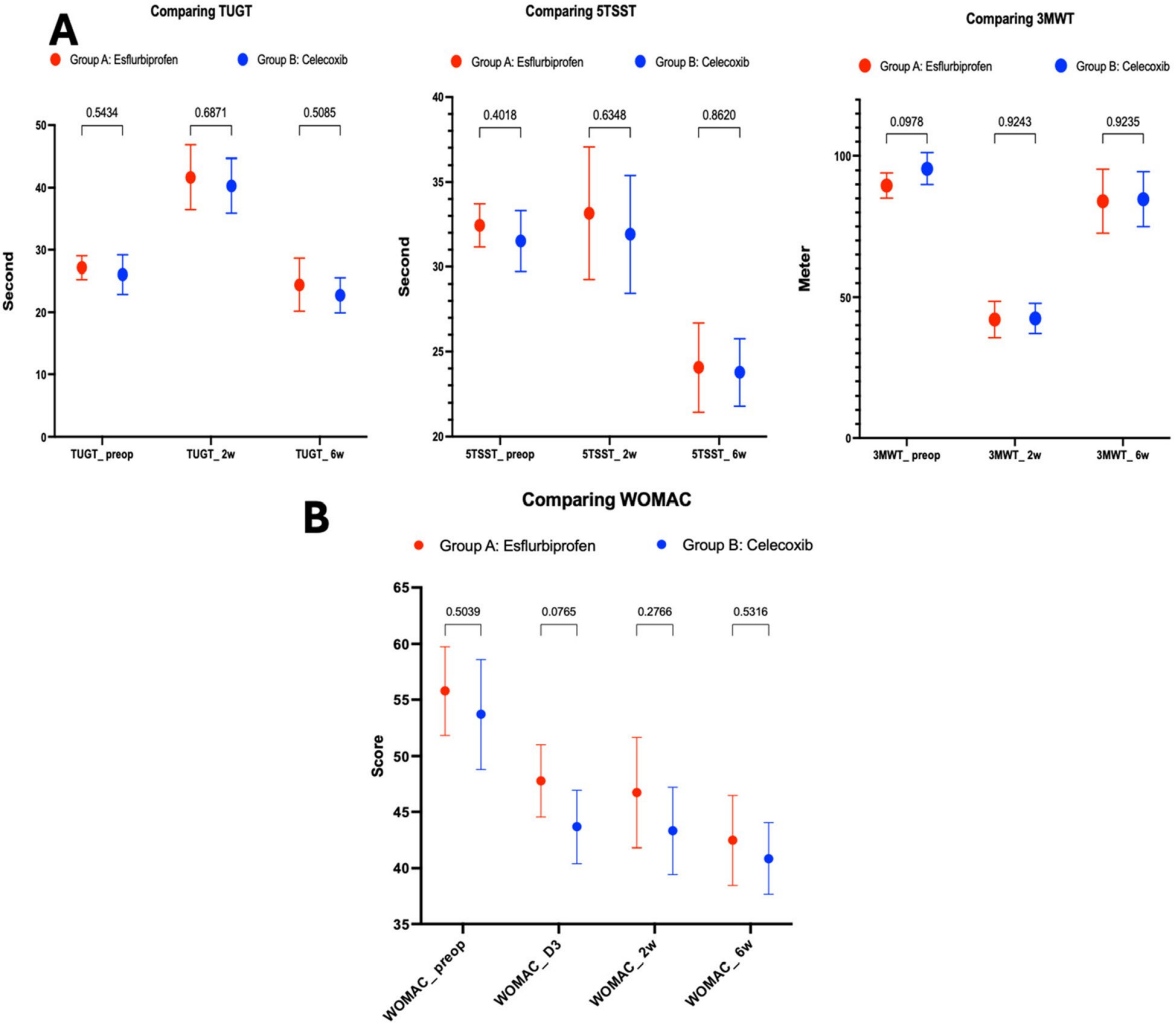
Comparing changes of time up-and-go test (TUGT), five-time sit-to-stand test (5TSST), three-minute-walk test (3MWT), and Western Ontario and McMaster Universities’ Osteoarthritis Index (WOMAC) at baseline, postoperative day 3, the 2nd week, and the 6th week between Group A: Esflurbiprofen and Group B: Celecoxib. **A**: Both groups had no differences in the changes of TUGT, 5TSST, and 3MWT at all points of evaluation. However, all tests were significantly worse at the 2nd week and improved at the 6th week. **B**: Both groups had similarly significantly improved WOMAC from POD 3 until the 6th week

Both groups had worse TUGT and 5TSST at POD 14, with gradually improving levels than at baseline at 6 weeks post-operatively. Similar 3MWT scores for both groups were worse at POD 14, but improvement at 6-week FU still did not return to baseline. Comparing all PBMs, including TUGT, 5TSST, and 3MWT, between the two groups, there were no differences at any time point: baseline, POD 14, and 6 weeks post-operatively, as shown in Fig. 4A. The WOMAC index in both groups was significantly improved from baseline at POD 3, POD 14, and 6 weeks post-operatively (Table 3); however, there were no differences in WOMAC index improvement between the two groups, as shown in Fig. 4B.

**Table 3:** Changes in WOMAC scores and GFR of both groups from baseline to the 6th week. Both groups had significantly improved WOMAC scores from postoperative day 3 (POD 3) through the 2nd week and at the 6th-week follow-up. There were no significant changes in GFR in either group from baseline through POD 1, the 2nd week, or the 6th-week follow-up

Parameter	Baseline vs different time points	Mean difference	Lower 95% CI	Upper 95% CI	*p*-Value
**WOMAC**	**Group A: Esflurbiprofen**				
	WOMAC_ preop vs. WOMAC_ D3	8.0	3.6	12.4	< 0.0001
	WOMAC_ preop vs. WOMAC_ wk2	9.1	0.5	17.6	0.0335
	WOMAC_ preop vs. WOMAC_ wk6	13.3	5.7	21.0	0.0002
	**Group B: Celecoxib**				
	WOMAC_ preop vs. WOMAC_ D3	10.0	5.2	14.8	< 0.0001
	WOMAC_ preop vs. WOMAC_ wk2	10.4	3.2	17.6	0.0019
	WOMAC_ preop vs. WOMAC_ wk6	12.8	5.9	19.8	< 0.0001
**GFR**	**Group A: Esflurbiprofen**				
	GFR_preop vs. GFR_D1	6.07	1.78	10.36	0.00
	GFR_preop vs. GFR_wk2	4.28	− 0.01	8.57	0.05
	GFR_preop vs. GFR_wk6	− 1.12	− 5.41	3.17	0.87
	**Group B: Celecoxib**				
	GFR_preop vs. GFR_D1	4.59	0.30	8.88	0.03
	GFR_preop vs. GFR_wk2	2.18	− 2.11	6.47	0.49
	GFR_preop vs. GFR_wk6	− 1.84	− 6.13	2.45	0.62

*WOMAC* Western Ontario and McMaster Universities Osteoarthritis Index, *GFR* glomerular filtration rate

**Fig. 5:**
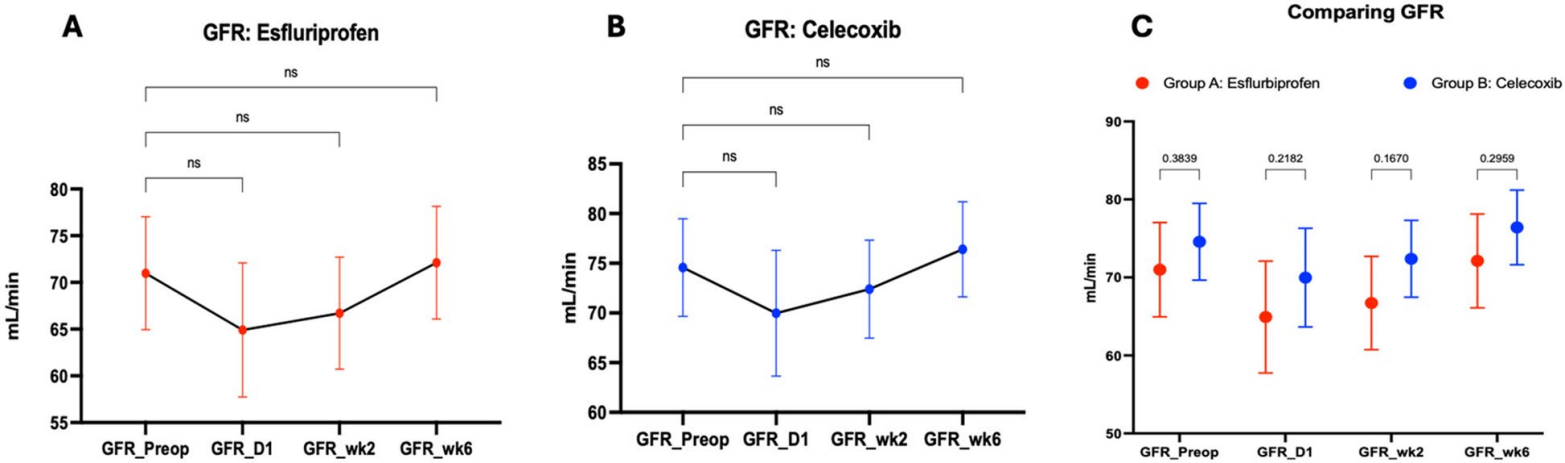
Graphs demonstrating and comparing changes in glomerular filtration rate (GFR) at baseline, on postoperative day 1, on the 2nd, and on the 6th week between Groups **A**: Esfluriprofen and **B**: Celecoxib. **A** and **B**: Both groups had no significant changes in the GFR at all points of evaluation. **C**: There were no differences in GFR at all points of evaluation between the two groups

Regarding the renal function evaluation using GFR, both Group A and B showed no change in GFR values from baseline at POD 1, POD 14, and 6 weeks post-operatively (Table 3), with no differences between the two groups (Fig. 5). There were no local skin or systemic adverse events that led to treatment discontinuation in either of the two studied groups.

## Discussion

The present RCT found that, on POD 14, transdermal Esflurbiprofen was noninferior to oral Celecoxib in VAS at rest and during motion after TKA. In addition, at 6 weeks post-operatively, the VAS trajectories, PBMs (TUGT, 5TSST, and 3MWT), and PROM (WOMAC) were comparable between the two treatment groups, and renal safety profiles were comparable as well, reinforcing the clinical equivalence of the two analgesic strategies.

Previous studies have consistently proven the efficacy of oral NSAIDs, of which Celecoxib is one among the commonly used oral NSAIDs, in reducing post-operative pain and opioid consumption following TKA, supporting its role as a cornerstone of multimodal analgesia regimens [6, 12, 22]. Although COX-2 inhibitors are the NSAIDs that effectively reduce gastrointestinal adverse events, they remain associated with potential systemic adverse effects, particularly with long-term use [23].

Topical NSAIDs, providing effective analgesic concentrations only at the site of action, have been available in the market for musculoskeletal pain for over 20 years [24]. From a pharmacokinetic and pharmacodynamic perspective, topical application significantly reduces systemic drug exposure and may be safer than oral NSAIDs, while adverse reactions most commonly involve cutaneous irritation, affecting only 2% of patients. Therefore, topical NSAIDs are increasingly used to treat acute and chronic musculoskeletal pain, with a recommendation to apply to patients who have a high risk of adverse events from oral NSAIDs.

Transdermal Esflurbiprofen, a topical NSAID, was launched in 2016 for the treatment of KOA, with reports of satisfactory efficacy following 12 h of application, attributed to its absorption into the deep tissues, synovium, and synovial fluid of the knee joint [25, 26]. Several clinical studies evaluating transdermal Esflurbiprofen patches demonstrated significant analgesic benefits compared with placebo in musculoskeletal pain conditions, with reduced systemic drug exposure [14, 15, 26, 27]. However, there were limited studies regarding the outcomes of topical NSAIDs on post-operative pain control after joint replacement.

A randomized study by Tsuji et al. comparing two transdermal patches (Flurbiprofen and Esflurbiprofen) reported that both agents provided effective pain relief and a favorable safety profile after TKA [28]. Another study by Tubsrinuan et al., comparing transdermal Esflurbiprofen with placebo for post-operative pain control after TKA, demonstrated significant reductions in pain scores after the first post-operative week, decreased morphine consumption, and improved functional scores [29]. Although both randomized studies reported positive outcomes for a topical NSAID in post-operative pain control after TKA, there have been no direct comparisons between topical NSAIDs and commonly used systemic oral COX-2 inhibitors in this population. To our knowledge, the present study is among the first to provide a head-to-head comparison of transdermal Esflurbiprofen and oral Celecoxib, directly addressing the clinical choice between local versus systemic NSAID delivery. Our findings indicate that transdermal Esflurbiprofen is not inferior to oral Celecoxib for immediate post-operative pain control, with similarly improved pain trajectories and functional recovery.

The present study design was aimed at evaluating post-operative pain control after TKA via an alternative option of NSAIDs from oral to topical administration. Therefore, it was an open-label trial, which might have introduced performance bias due to the different drug administration routes. Patients receiving a local skin patch might experience a positive placebo effect with a new agent with a new route of administration, whereas patients receiving oral medication might not. However, independent assessors were assigned to record the VAS and WOMAC scores at all time points via the interview. In addition, using PBMs, including 5TSST, TUGT, and 3MWT, reflected patients’ actual abilities, thereby reducing observer bias.

Unlike previous studies reporting that cutaneous adverse reactions could occur in 1 to 2% of patients using topical NSAIDs, including erythema, pruritis, irritation, sensation of heat or burning, and contact dermatitis [24, 28], the present study did not observe this adverse event in patients in Group A (transdermal Esflurbiprofen). Similarly, all patients in group B (oral Celecoxib) experienced no adverse reactions. The limited duration of medication administration in both groups, up to 14 days, might be related to the limited number of adverse events. As changes in renal function, monitored by serial GFR at different time points, did not differ between patients in both groups, transdermal Esflurbiprofen could be considered to have a safety profile similar to that of oral Celecoxib.

Currently, opioid-sparing pain management in TKA using single-shot or continuous multiple nerve blocks, including adductor canal block (ACB), interspace between popliteal artery and posterior capsule of the knee (iPACK) block, local infiltration anesthesia (LIA), as well as multiple combined medications, has been proven to provide very efficient pain control without adverse events to delay early mobilization [20, 21, 30]. According to our previous studies, the efficacy of multimodal opioid-sparing post-operative pain control provided satisfactory pain levels with the use of a tourniquet at the time of surgery [20, 21, 30]. The VAS at rest and on motion on POD 0, POD 1, POD 2, and POD 3, as well as the WOMAC index on POD 3, in patients in both groups in the present study, confirmed the efficacy of opioid-sparing pain control after TKA. However, with a combination of anesthesia and several medications during the perioperative period, we could not claim a positive effect of transdermal Esflurbiprofen or Celecoxib for pain and functional improvement during hospital admission.

While peri-operative pain control with early recovery after surgery (ERAS) during hospital admission resulted in very high patient satisfaction, increasing immediate-to-subacute pain after discharge is still problematic [31]. Several studies have demonstrated that the intensity of Immediate-to-subacute pain during 1 to 3 months after TKA is predictive of 1-year satisfaction [32–34]. Oral NSAIDs are one of the important multimodal pain control agents prescribed as a home medication [35]. While Celecoxib, a COX-2 inhibitor, is commonly selected as a pain control agent for home medications, some patients experienced adverse events, with the gastrointestinal system being the most common [18, 36, 37]. Therefore, having an alternative option of NSAIDs as home medication could provide more choice for those who are concerned about adverse events from the oral form. The topical NSAID, as an alternative to an oral one used in the present study, was expected to reduce the common adverse events from oral NSAIDs; however, we did not observe any significant difference in adverse events between the two agents.

This study had some limitations. Firstly, although the present study was powered primarily for peri-operative and immediate pain outcomes after TKA, a relatively short duration of drug administration might not identify the actual adverse events of both agents. Further study with a larger sample size and an extended treatment duration may provide more information. Secondly, although outcome assessors were blinded, participants’ blinding was limited by differences in the drug administration route. However, this study design was aimed at evaluating post-operative pain control after TKA via an alternative option of NSAIDs from systemic to topical administration. Further study with a double-dummy design, a larger sample size, and an extended treatment duration may provide more information and eliminate the pharmacological effect from the psychological impact of the administration route.

## Conclusion

A multimodal pain control regimen in TKA using a transdermal Esflurbiprofen patch provided peri-operative and immediate post-operative pain control that was non-inferior to oral Celecoxib, with comparable VAS, functional outcomes, and safety profiles. Topical NSAID delivery represented a feasible alternative to systemic therapy, particularly for patients in whom systemic NSAID exposure is undesirable.

## Data Availability

The dataset supporting the conclusion of this article is available upon request from the corresponding author (AT and SN).
